# Revealing the Hidden Severity: A Case Report on Managing Complex Aortic Stenosis With TAVI

**DOI:** 10.1155/cric/1945506

**Published:** 2026-06-03

**Authors:** Thomas Stefoulis, James Sindone, Mahiar Mahjoub, Samuel Jay-Jackson Pack, Sean Lal

**Affiliations:** ^1^ Junior Medical Workforce, Royal Prince Alfred Hospital, Sydney, New South Wales, Australia, nsw.gov.au; ^2^ Cardiology Department, Royal Prince Alfred Hospital, Sydney, New South Wales, Australia, nsw.gov.au; ^3^ Junior Medical Workforce, Bankstown Hospital, Sydney, New South Wales, Australia

**Keywords:** Agatston score, aortic stenosis, case report, multimodal diagnostic approach, TAVI, valvular disease

## Abstract

Aortic stenosis (AS) is a progressive condition prevalent in older adults, with severity determined through clinical evaluation and imaging. However, diagnostic dilemmas arise when imaging results conflict with clinical symptoms. This report examines an 84‐year‐old male with chronic kidney disease, prior abdominal aortic aneurysm repair and other comorbidities, presenting with decompensated heart failure. Initial echocardiography findings suggested moderate AS, insufficient to qualify for transcatheter aortic valve implantation (TAVI) under national guidelines, despite clinical evidence of worsening heart failure. A multimodal diagnostic approach, incorporating Agatston scoring, dobutamine stress echocardiography (DSE), and invasive haemodynamics, was undertaken to resolve this discordance. DSE demonstrated persistent discordance between Doppler‐ and Simpson‐derived flow estimates, whereas CT calcium scoring and left heart catheterisation ultimately confirmed severe AS, with an aortic valve area of 0.75 cm^2^. The patient underwent preoperative optimisation, including multidisciplinary team (MDT) input and targeted interventions to manage comorbidities and optimise haemodynamics. TAVI was performed successfully, resulting in significant improvements in symptoms, exercise tolerance and renal function. This case highlights the necessity of a multimodal diagnostic strategy when initial investigations do not align with clinical presentation, emphasising MDT collaboration and preoperative optimisation. It demonstrates how multimodality imaging can prevent underestimation of AS severity and support timely intervention in clinically complex patients.

**Learning Objectives**

This case underscores the importance of a multimodal diagnostic approach to confirm aortic stenosis (AS) severity when clinical symptoms and initial investigations are discordant. It highlights the value of integrating transthoracic echocardiography, dobutamine stress echocardiography (DSE), CT calcium scoring and invasive haemodynamics to guide management. It also emphasises the role of preoperative optimisation and multidisciplinary team (MDT) input in improving outcomes for high‐risk patients considered for TAVI.

## 1. Introduction

Valvular AS is a common clinical entity, affecting up to 10% of 80–89‐year‐olds, the prevalence of which increases exponentially with age [1]. AS comprises a spectrum of disease states, ranging from mild asymptomatic to severe symptomatic. AS is a progressive condition, with symptoms of dyspnoea and angina generally correlating with disease severity. Complications include heart failure, pulmonary hypertension, arrhythmia and sudden cardiac death.

Diagnosis of AS is a stepwise process integrating clinical evaluation, examination and a series of targeted diagnostic tests. Echocardiography remains the cornerstone, but additional tests including cardiac CT, left heart catheterisation (LHC), and DSE may be required when imaging findings are discordant with the clinical presentation, especially where clinical severity is incongruent with imaging severity [2].

Transcatheter aortic valve implantation (TAVI) has become the treatment of choice for severe symptomatic AS [3] in the elderly or those who may be deemed of increased surgical risk. The severity of AS is evaluated primarily via resting transthoracic echocardiogram (TTE) correlated to patient symptoms. Unfortunately, due to the high prevalence of AS in the elderly, symptomatology can be confounded by patient comorbidities, whereas technical difficulties may be encountered on echocardiography in measuring the aortic valve area (AVA) or aortic valve gradient. Therefore, a multimodal and multidisciplinary approach to the evaluation of AS severity is critical in determining which patients would benefit from TAVI.

We report the case of an 84‐year‐old man with recurrent decompensated heart failure in whom AS severity remained uncertain because imaging findings were discordant with his clinical deterioration.

### 2. Case Presentation

The patient is an 84‐year‐old male of European heritage with a complex medical history, who presented to a tertiary university teaching hospital in Sydney, Australia with decompensated heart failure and suspected clinically significant AS. His past medical history included stage IV chronic kidney disease as per KDIGO guidelines [4], endovascular abdominal aortic aneurysm repair in 2019, moderate AS on echocardiography, dual chamber permanent pacemaker for tachy‐brady syndrome (and hence chronotropic incompetence), right upper lobe lobectomy in 2019 for lung cancer (now in remission), right carotid endarterectomy for carotid stenosis in 2019 and latent tuberculosis. These factors elevated his surgical risk, making traditional surgical aortic valve replacement less favourable [5].

These comorbidities translated into very high predicted surgical risk. A EuroSCORE II calculation estimated an in‐hospital mortality of 32.7%, and the Society of Thoracic Surgeons (STS) risk calculator predicted an operative mortality of 23.2% for combined coronary artery bypass grafting and aortic valve replacement. Together, these scores placed him in a very high‐risk category for surgical valve replacement.

The patient was independent with activities of daily living and had no cognitive impairment. His exercise tolerance had deteriorated to five metres in the preceding 3 months, correlating with three hospital admissions for decompensated heart failure within 6 months. His medication regimen included amlodipine, aspirin, atorvastatin, clopidogrel, furosemide, metoprolol and oxazepam.

The patient presented with decompensated heart failure with fluid overload. Clinical assessment revealed elevated jugular venous pressure, bilateral pulmonary infiltrates and peripheral oedema to mid‐shins. Heart sounds were notable for a harsh, late‐peaking ejection systolic murmur radiating to the carotids.

**Figure 1:**
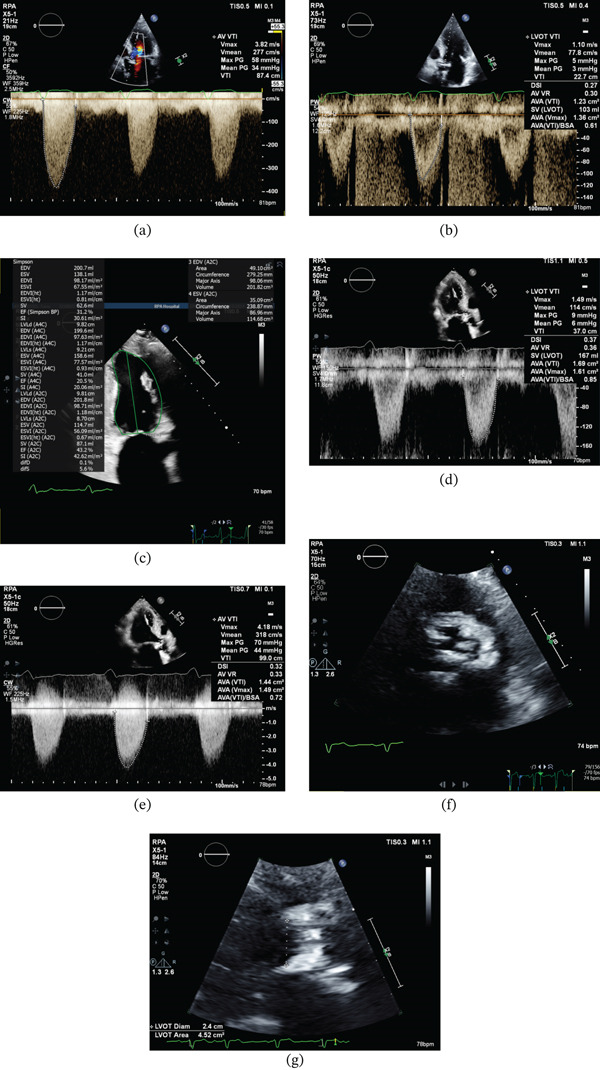
Available transthoracic and dobutamine stress echocardiographic images demonstrating discordant low‐gradient aortic stenosis. (a) Baseline/rest continuous‐wave Doppler across the aortic valve, showing Vmax 3.82 m/s, mean gradient 34 mmHg, and VTI 87.4 cm on a representative beat. (b) Baseline/rest LVOT pulsed‐wave Doppler, showing VTI 22.7 cm and Doppler‐derived stroke volume 103 mL on a representative beat. (c) Peak‐stress apical biplane Simpson assessment, showing SVi 30.61 mL/m^2^ and LVEF 31.2% on a representative frame. (d) Peak‐stress LVOT pulsed‐wave Doppler, showing VTI 37.0 cm and Doppler‐derived stroke volume 167 mL on a representative beat. (e) Peak‐stress continuous‐wave Doppler across the aortic valve, showing Vmax 4.18 m/s, mean gradient 44 mmHg, and continuity‐equation AVA 1.44–1.49 cm^2^ on a representative beat. (f) Two‐dimensional short‐axis transthoracic echocardiographic view demonstrating marked calcification of the aortic valve. (g) LVOT diameter measurement view demonstrating an LVOT diameter of 2.4 cm and LVOT area of 4.52 cm^2^. Representative still frames are shown. Displayed overlay values may differ slightly from the tabulated study‐summary values cited in the text because of beat‐to‐beat variability and tracing differences.

TTE demonstrated severe aortic valve calcification with a mean systolic gradient over the aortic valve of 32 mmHg, a left ventricular ejection fraction (LVEF) of approximately 35%–40% (38% by Simpson′s biplane method), and a moderate calculated AVA of 1.05–1.1 cm^2^. LV stroke volume index (SVi) by Simpson′s biplane method was 26.5 mL/m^2^, consistent with a low‐flow state. In contrast, left ventricular outflow tract (LVOT) Doppler‐derived SVi was 44 mL/m^2^, which is not within the low‐flow range. This discrepancy raised concern that LVOT Doppler may have overestimated forward flow, particularly in the setting of a heavily calcified subannular/LVOT region. Aortic valve Doppler showed peak and mean gradients of 56 and 32 mmHg, respectively, corresponding to a peak jet velocity of approximately 3.8 m/s, with an LVOT/AV VTI ratio of 0.23. Doppler envelopes were obtained from multiple acoustic windows, including apical and right parasternal views to minimise underestimation of peak aortic velocity. However, heavy calcification of the sub‐annular part of the aortic valve led to technical difficulties in accurately measuring the LVOT area. Baseline AV continuous‐wave Doppler and LVOT pulsed‐wave Doppler are shown in Figure 1a,b, respectively; marked aortic valve calcification and the LVOT diameter measurement are shown in Figure 1f,g.

Pre‐procedural multidetector CT (MDCT) performed for TAVI planning in 2021 demonstrated an annular/LVOT cross‐sectional area of 4.56 cm^2^ (diameter Dmin 22.2 mm, Dmax 29.0 mm, derived circular diameter 24.1 mm, circumference 78.5 mm). This CT‐derived LVOT area closely matched the echocardiographic LVOT area of 4.5 cm^2^ (diameter 24 mm), suggesting that the discordance was unlikely to be explained solely by gross LVOT area overestimation, although this did not exclude possible overestimation of LVOT Doppler‐derived flow. This also effectively provided a hybrid MDCT–Doppler geometric assessment without the need for 3D TOE (Transoesophageal echocardiography), which was avoided due to the patient′s anaesthetic risk. Thus, the patient was not initially offered a TAVI due to these findings not meeting severity criteria under Australian guidelines, with concerns raised regarding ‘pseudo‐severe’ AS, although low‐flow low‐gradient, was not considered by the structural heart team as a potential cause of symptomatology.

Given our concerns regarding the patient′s clinical severity of AS, we proceeded to Agatston scoring of the aortic valve which gave a score of 2780, consistent with severe aortic valve calcification and supportive of severe AS [6].

DSE provided further haemodynamic clarification. At peak stress, transaortic velocity and gradients increased, with peak aortic jet velocity rising to 4.2 m/s and mean/peak gradients increasing to 44/71 mmHg. However, there was marked discordance between Simpson biplane‐derived and LVOT Doppler‐derived estimates of stroke volume. Simpson biplane‐derived SVi increased only modestly from 26.5 to 30.6 mL/m^2^, whereas LVOT Doppler‐derived SVi increased from 44 to 77 mL/m^2^. The continuity‐equation AVA increased to 1.6 cm^2^, remaining in the moderate range despite the high transvalvular gradients. On retrospective review, this discrepancy raised concern that LVOT Doppler flow may have been overestimated at peak stress, potentially related to upper septal hypertrophy and/or LVOT flow acceleration. Accordingly, the DSE findings were interpreted with caution, and CT calcium scoring together with invasive haemodynamics were used to adjudicate AS severity. Representative peak‐stress DSE images are shown in Figure 1c–e, demonstrating apical biplane Simpson assessment, LVOT pulsed‐wave Doppler, and aortic valve continuous‐wave Doppler, respectively. Key multimodality measurements are summarised in Table 1.

**Table 1:** Multimodality assessment of aortic stenosis severity.

Modality	Key Measurements	Interpretation
Baseline TTE	Simpson SVi 26.5 mL/m^2^; LVOT Doppler SVi 44 mL/m^2^; mean/peak gradient 32/56 mmHg; Vmax ~ 3.8 m/s; AVA 1.05–1.1 cm^2^; LVOT/AV VTI ratio 0.23	Low flow by Simpson method with discordant Doppler‐derived flow and AVA
DSE	Simpson SVi 30.6 mL/m^2^; LVOT Doppler SVi 77 mL/m^2^; mean/peak gradient 44/71 mmHg; Vmax 4.2 m/s; AVA 1.6 cm^2^	Higher gradients and velocity but persistent AVA discordance; possible LVOT Doppler overestimation
MDCT	Agatston score 2780; LVOT/annular area 4.56 cm^2^; Dmin 22.2 mm; Dmax 29.0 mm; circumference 78.5 mm	Severe valve calcification; hybrid geometric confirmation
LHC	AVA 0.75 cm^2^; peak/mean gradient 46/39 mmHg	Invasive confirmation of severe AS

Coronary angiography demonstrated 80% stenosis in the proximal left anterior descending (LAD) artery and 60% stenosis in the second diagonal artery, necessitating percutaneous coronary intervention to the LAD for preprocedural optimisation. A concurrent LHC confirmed severe AS with an AVA of 0.75 cm^2^ and peak/mean transaortic gradients of 46/39 mmHg, and the patient subsequently met severity criteria for TAVI implementation, which was promptly scheduled.

PreTAVI optimisation included dobutamine and furosemide infusions to increase forward flow to the kidneys, resulting in significant diuresis and clinical improvement. A fluid restriction of 1 L/day was instituted, and he received intravenous iron. After cessation of dobutamine and furosemide, levosimendan was administered to optimise haemodynamics prior to TAVI. This was coordinated jointly by the Structural Heart and Renal teams, with close monitoring of renal function and volume status.

On 11th of April 2024, the patient successfully underwent a TAVI with a Medtronic Evolut 29 mm valve deployed via the right femoral artery. There was no significant aortic regurgitation postprocedure. The procedure was performed under general anaesthesia with close haemodynamic monitoring. Immediately postoperatively, the patient reported significant subjective clinical improvement in symptom burden. He was able to self‐mobilise over 100 metres without dyspnoea, with improved functional status to levels not previously experienced for several years. His kidney function improved significantly with a doubling of his eGFR. Ongoing improvement in exercise tolerance was reported on postoperative follow‐up with ongoing engagement with the Cardiac Rehabilitation Team.

## 3. Discussion

The patient presented with a complex clinical picture of heart failure and renal failure, progressing from moderate to severe AS, ultimately TAVI. Despite his initial presentation not entirely meeting criteria for severe AS based on static echocardiographic measurements alone, his symptoms and progressive heart failure suggested a more critical clinical course.

Given the patient′s advanced age, comorbidities and symptomatic presentation, accurately diagnosing the severity of his AS was critical. According to contemporary guideline‐based assessment [5], low flow is defined by SVi ≤ 35 mL/m^2^, and DSE can help distinguish true severe from pseudo‐severe AS in low‐flow, low‐gradient AS with reduced LVEF. In this patient, the LVOT and subannular region were heavily calcified, making 2D measurement of the LVOT diameter challenging. The LVOT diameter on TTE was 24 mm (area 4.5 cm^2^), which closely matched the cross‐sectional area of 4.56 cm^2^ obtained from preprocedural MDCT, arguing against gross overestimation of LVOT area. However, on retrospective review of the available DSE images, mild upper septal hypertrophy and/or LVOT flow acceleration at peak stress could not be excluded. This may have contributed to overestimation of LVOT Doppler‐derived stroke volume and, consequently, continuity‐equation AVA. Multiple Doppler windows, including the right parasternal view, were used to minimise underestimation of AV velocity; baseline peak aortic jet velocity was approximately 3.8 m/s with peak/mean gradients of 56/32 mmHg. Despite these precautions, the calculated AVA remained in the moderate range, illustrating how technical limitations in AVA calculation can still lead to discordant grading.

The mismatch between symptoms and imaging prompted further evaluation using additional diagnostic modalities, as recommended by the ESC/EACTS guidelines [2, 5]. Hybrid approaches that combine CT‐derived LVOT area with Doppler haemodynamics are recommended when 2D echocardiographic LVOT measurements are unreliable. In our case, the near‐identical LVOT area by MDCT (4.56 cm^2^) and TTE (4.5 cm^2^) suggested that the discordance was unlikely to be explained solely by gross LVOT area overestimation. However, this did not exclude possible overestimation of LVOT Doppler‐derived flow during DSE, which remained a plausible contributor to the discordant echocardiographic results.

DSE is useful in low‐flow, low‐gradient AS because it provides information on how transvalvular velocity, gradient and valve area change as flow increases [7]. Contemporary data suggest that, during DSE, peak velocity and mean gradient may outperform AVA for identifying severe AS, although diagnostic performance varies across LVEF strata [8]. In our case, DSE showed higher transvalvular velocities and gradients, with Vmax increasing to 4.2 m/s and mean gradient to 44 mmHg. However, the marked discrepancy between Simpson biplane‐derived and LVOT Doppler‐derived SVi raised the possibility that LVOT Doppler overestimated forward flow at peak stress. This may explain why the continuity‐equation AVA remained in the moderate range despite high transvalvular gradients, further underscoring the need for multimodality confirmation with CT calcium scoring and invasive haemodynamics.

CT calcium valve scoring was used because this modality provides a flow‐independent assessment of aortic valve calcification and is particularly helpful when echocardiographic findings are ambiguous [6]. The patient′s Agatston score of 2780 was supportive of severe AS and reinforced the decision to proceed with TAVI.

LHC ultimately provided invasive confirmation of severe AS in our case, with an AVA of 0.75 cm^2^ and peak/mean gradients of 46/39 mmHg. Although invasive haemodynamics are not required routinely, LHC remains an important adjudicative test when noninvasive findings are inconclusive [2, 5]. Potential complications of cardiac catheterisation include vascular injury, arrhythmia, stroke, infection and adverse reactions to contrast or medications [9].

Although well‐defined echocardiographic parameters categorise AS severity, discordant findings are common and warrant further evaluation with DSE and/or CT calcium scoring in appropriate cases [2, 10]. Low‐gradient AS commonly leads to discordant severity grading. DSE is recommended primarily in patients with classic low‐flow, low‐gradient AS and reduced LVEF when there is concern regarding underestimation of severity, whereas noncontrast CT calcium scoring provides a flow‐independent assessment of valve calcification across low‐gradient AS phenotypes [6, 10]. This rationale underpinned our decision to obtain an Agatston score in this patient.

In summary, this case illustrates how severe symptomatic low‐flow, low‐gradient AS may be underestimated when resting AVA and gradient are discordant and LVOT Doppler measurements are potentially misleading. In this patient, CT calcium scoring and invasive haemodynamics were decisive in confirming severe AS and supporting TAVI, whereas preprocedural coronary intervention and heart failure optimisation enabled a successful outcome. A limitation of this report is that not all archived multimodality still‐frame images and invasive tracings were retrievable in publication‐quality form; however, the available echocardiographic images and the quantitative data summarised in Table 1 were sufficient to demonstrate the diagnostic discordance and the rationale for multimodality adjudication. The completed CARE checklist is provided as Supporting Information 1 (available here).

**Nomenclature**
AS: aortic stenosis
AVA: aortic valve area
DSE: dobutamine stress echocardiography
LHC: left heart catheterisation
LVOT: left ventricular outflow tract
MDCT: multidetector computed tomography
SVi: stroke volume index
Vmax: peak transvalvular velocity
VTI: velocity‐time integral.

## Author Contributions

Thomas Stefoulis: Primary author, assisted in clinical case management, data collection, manuscript writing and revision. James Sindone: Assisted in clinical case management, manuscript drafting and provided critical feedback. Mahiar Mahjoub: Assisted in clinical case management and summary figure preparation. Samuel Jay‐Jackson Pack: Contributed to the manuscript. Sean Lal: Supervised the case report, provided expert guidance, and reviewed the manuscript.

## Funding

No funding was received for this manuscript.

## Consent

Consent for this case report was obtained from the patient and his next of kin on 10/05/2024. Both hope that this story may assist others in receiving timely intervention.

## Conflicts of Interest

The authors declare no conflicts of interest.

## Supporting information


**Supporting Information** Additional supporting information can be found online in the Supporting Information section. CARE Checklist. Completed CARE checklist for this case report, outlining adherence to the CARE case report reporting guidelines.

## Data Availability

The data that support the findings of this study are available from the corresponding author upon reasonable request.
