# Supplementary material for: Revealing the Hidden Severity: A Case Report on Managing Complex Aortic Stenosis With TAVI
Source: Case Rep Cardiol. 2026 Jun 3;2026:1945506. doi: 10.1155/cric/1945506 (PMC13239360; doi:10.1155/cric/1945506)
Supplement: Supplementary file 1 — Supporting Information Additional supporting information can be found online in the Supporting Information section. CARE Checklist. Completed CARE checklist for this case report, outlining adherence to the CARE case report reporting guidelines. [file CRIC-2026-1945506-s001.pdf]

CARE CHECKLIST FOR EHJ-CR

Topic	Item	Checklist item description	Author Confirmation	Reviewer Confirmation
Title	1	The words "case report" should be in the title along with the area of focus	✔	
Key Words	2	Four to seven key words—including "case report" as a key word	✔	
Abstract	3a	Background: What does this case report add to the medical literature	✔	
	3b	Case summary (1 paragraph): chief complaint, diagnoses, interventions, and outcomes	✔	
	3c	Conclusion: What are the main "take-away" lessons from this case?	✔	
Introduction	4	How does this case informs healthcare delivery—with references (1-2 paragraphs)	✔	
Timeline	5	Relevant information from this case report organized into a timeline (table or figure)	✔	
Patient Information	6a	De-identified demographic and other patient specific information	✔	
	6b	Chief complaint (what prompted this patient visit)	✔	
	6c	Relevant medical and psychosocial history (including interventions and outcomes)	✔	
Physical Exam	7	Relevant physical examination findings	✔	
Diagnostic Assessment	8a	Diagnostic evaluations (such as laboratory testing, imaging, surveys)	✔	
	8b	Diagnoses (consider tables/figures linking assessment with diagnoses and interventions)	✔	
	8c	Diagnostic reasoning including other diagnoses considered and diagnostic challenges	✔	
	8d	Prognostic characteristics (such as staging in oncology) where applicable	✔	
Interventions	9a	Types of intervention (such as pharmacologic, surgical, preventive, self-care)	✔	
	9b	Intervention administration (such as dosage, strength, duration)	✔	
	9c	Changes in intervention (with rationale)	N/A	
	9d	Other concurrent interventions	N/A	
Follow-up and Outcomes	10a	Clinician and patient-assessed outcomes (when appropriate)	✔	
	10b	Important follow-up diagnostic evaluations	✔	
	10c	Assessment of intervention adherence and tolerability	✔	
	10d	Adverse and unanticipated events	✔	
Discussion	11a	Strengths and limitations in your approach to this case—with references	✔	
	11b	Conclusions and rationale (including possible causes for outcomes)	✔	
Patient Perspective	12	When appropriate include the patient's perspective on this episode of care	N/A	
Informed Consent	13	Patient informed consent is likely to be required by a journal (or your institution) prior to publication	✔	
Other	14	IRB approval as indicated or needed;<br>Acknowledgement section;<br>Competing Interests;<br>Funding	✔	

Adapted from http://data.care-statement.org/
